# Supplementary material for: Estrogen receptor 1 gene polymorphisms (PvuII and XbaI) are associated with type 2 diabetes in Palestinian women
Source: PeerJ. 2019 Jun 28;7:e7164. doi: 10.7717/peerj.7164 (PMC6601601; doi:10.7717/peerj.7164)
Supplement: Supplemental Information 1 [file peerj-07-7164-s001.docx]

Table S1: Primers and virtual probes used in PCR and sequence analysis

|  | | |
| --- | --- | --- |
| **Primer name** | **Primer sequence including Illumina adaptor in bold** | **PCR Specificity** |
| EstFNGS | ***TCGTCGGCAGCGTCAGATGTGTATAAGAGACAG***CTGTGTTGTCCATCACTTCATC | ESTR1 forward |
| EstRNGS | ***GTCTCGTGGGCTCGGAGATGTGTATAAGAGACAG***CCATTAGAGACCAATGCTCATC | ESTR1 Reverse |
| **Probe Name** | **Probes sequences-SNP is in bold** | **Probe specificity** |
| rs9340799c | TGTCCCAGC**C**GTTTTATGCT | C |
| rs9340799t | TGTCCCAGC**T**GTTTTATGCT | T |
| rs2234693g | GTGTGGTCT**G**GAGTTGG | G |
| rs2234693a | GTGTGGTCT**A**GAGTTGG | A |
